# Evaluation of Methods for Sampling of *Staphylococcus aureus* and Other *Staphylococcus* Species from Indoor Surfaces

**DOI:** 10.1093/annweh/wxaa080

**Published:** 2020-09-24

**Authors:** Anne Mette Madsen, Hoang U T Phan, Mathias Laursen, John K White, Katrine Uhrbrand

**Affiliations:** National Research Centre for the Working Environment, Lersø Parkallé, Copenhagen Ø, Denmark

**Keywords:** *Corynebacterium*, environmental contamination, fomite sampling, MALDI-TOF MS, methicillin resistance, replicate sampling, sedimented dust, *Staphylococcus aureus*, *Staphylococcus* diversity, surface sampling, swabs

## Abstract

**Objectives:**

Methicillin-resistant *Staphylococcus aureus* (MRSA) is an increasing public and occupational health concern. As transmission of MRSA can occur via contact with fomites, it is crucial to have sensitive methods for sampling of bacteria. The overall aim of this study was to obtain knowledge about methods and strategies for quantitative sampling *Staphylococcus* species on surfaces.

**Methods:**

The study was designed as a comparative sampling experiment with different samplers [dipslide (two agar types), swabs (three brands, used wet and dry, and elution from swabs or plate diluted)] on smooth stainless steel surfaces spiked with MRSA and methicillin-sensitive *S. aureus* (MSSA). Furthermore, bacteria sampled from indoor surfaces with frequent or infrequent contact with hands were quantified and identified using matrix-assisted laser desorption-ionization time-of-flight (MALDI-TOF) mass spectrometry (MS).

**Results:**

Pre-moistened swabs in combination with dilution plating and dipslides were more sensitive than dry swabs. For recovery of MRSA and MSSA from surfaces with eSwabs, at least 0.3–100 CFU MRSA cm^−2^ and 5.3–8.6 CFU MSSA cm^−2^ should be present. The sensitivities of pre-moistened eSwabs were approximately 10-fold higher than those of dipslides and pre-moistened viscose and cotton swabs. The variation in concentrations of *Staphylococcus* species in replicate sampling of adjacent squares on indoor surfaces was higher for surfaces frequently touched by hands than for surfaces infrequently touched. In total 16 different *Staphylococcus* species were identified, and *S. aureus* was found only in 2 of 66 surface samples. A considerable overlap was found between species in replicate sampling within an environment and between the air and surfaces within an environment.

**Conclusions:**

Pre-moistened eSwabs in combination with dilution plating were found to be the best method for surface sampling of MSSA and MRSA. The method can be used for assessing the risk of exposure and transmission of MRSA from environmental surfaces. To obtain a reliable measure of concentrations and the presence of *Staphylococccus* species a higher number of samples should be taken from surfaces with hand contact than from surfaces dominated by sedimented bacteria.

## Introduction

*Staphylococcus aureus* is an important and versatile opportunistic human pathogen with the ability to colonize individuals and cause superficial to moderate infections of skin and soft tissue or systemic and life-threatening conditions such as endocarditis, pneumonia, and bacteraemia (Aires de Sousa and Lencastre, 2004; Hogan *et al.*, 2015). Other *Staphylococcus* species such as *S*. *epidermidis*, *S*. *lugdunensis*, *S*. *saprophyticus*, and *S*. *haemolyticus* are less virulent than *S*. *aureus* (Rosenstein and Götz, 2012). However, they can cause occupational-, nosocomial-, and community-acquired infections (Rogers *et al*., 2009; Becker *et al.*, 2014), and they may play a role in horizontal transfer of antimicrobial resistance genes (Davis *et al.*, 2012; Mkrtchyan *et al*., 2013), and some species are commonly found in indoor air and sedimented dust (Madsen *et al.*, 2018).

Methicillin-resistant *S. aureus* (MRSA) are a serious concern, as infections are difficult to treat. Whereas MRSA infections were previously found to primarily affect individuals in healthcare facilities, occupational- and community-associated MRSA infections affecting healthy individuals with little or no connection to healthcare facilities are now frequently reported, increasing the risk of MRSA contamination and transmission occurring outside hospitals (Otter and French, 2009; Gupta *et al.*, 2015; Girbig *et al.*, 2017; Laustsen and Omland, 2019). Currently, many MRSA-positive individuals are treated outside of the hospitals e.g. in nursing homes and private residences. In these environments, MRSA-positive occupants are not necessarily in isolation. Therefore, we expect that transmission of MRSA may be of concern for employees and other occupants in e.g. nursing homes and private homes with MRSA-positive occupants.

The transmission of MRSA can occur directly from person-to-person or indirectly via contact with fomites. MRSA can be difficult to eliminate from the environment and can survive for several weeks on surfaces (Huang *et al.*, 2006; Kramer *et al.*, 2006) and in dust (Feld *et al.*, 2018). It has previously been detected on surfaces in hospital rooms with MRSA-colonized patients (Sexton *et al.*, 2006), in common areas of hospitals (Faires *et al.*, 2012), and in homes of MRSA-colonized individuals (Allen *et al.*, 1997; Davis *et al.*, 2012). In a hospital environment, the highest concentrations of aerobic bacteria in general have been found on surfaces that are regularly touched by patients or healthcare workers (Moore *et al.*, 2013), indicating that the primary source of the bacteria in this environment is human contact rather than sedimented dust. Thus, MRSA has been detected on a variety of different fomites, such as dirty laundry, sinks, toilet seats, television remote controls, and door handles (Davis *et al.*, 2012). Moreover, 36% of examined public buses in Lisbon have previously been shown to be contaminated with MRSA on surfaces with a high level of hand contact (Conceição *et al.*, 2013). Knowledge about the distribution of MRSA and other *Staphylococcus* species on surfaces is of importance in relations to potential transmission routes to patients and employees, but also in the planning of sampling strategies.

A variety of methods have been used for environmental sampling of MRSA and other bacteria on surfaces. Some studies employ various forms of contact plates with different agar types (Bartels *et al.*, 2008; Otter and French, 2009; Creamer *et al.*, 2014), while others use different types of swabs either in combination with direct swab incubation on agar (Boyce *et al.*, 1997) or by dilution plating often following an enrichment step (Creamer *et al.*, 2014; Visalachy *et al.*, 2016). Finally, some studies have performed surface sampling using electrostatic cloths (Faires *et al.*, 2012) or gauze (Conceição *et al.*, 2013). However, the efficacy of most of these methods are unknown and only a few studies have so far compared the efficacy of different surface sampling methods to recover methicillin-sensitive *S. aureus* (MSSA) (Landers *et al.*, 2010; Lutz *et al.*, 2013; Hogan *et al.*, 2015) and MRSA (Dolan *et al.*, 2011). To contribute to a better understanding of the presence and survival of MRSA on different kinds of surfaces, and to the extent to which surfaces play a role in the transmission of MRSA in various settings, e.g. hospitals, nursing homes, homes, and offices, an efficient and standardized method for surface sampling of MRSA allowing better comparison between studies is warranted. To enable quantitative microbial risk assessment (QMRA), such a method should ideally be compatible with quantitative detection of MRSA on the surfaces.

The overall aim of this study was to obtain knowledge about methods and strategies for quantitative sampling *Staphylococcus* species on surfaces. Our objective was to identify the most sensitive and robust sampling method for quantitative culture-based recovery of MRSA and MSSA from environmental surfaces. This was done by comparing sampling methods to recover MRSA and MSSA from artificially spiked surfaces and from office desk surfaces. To obtain knowledge about strategies for sampling, concentrations and species compositions of *Staphylococcus* on adjacent indoor surfaces with frequent or infrequent contact with hands were measured. Air samples, as a potential source of bacteria to surfaces with infrequent hand contact, were collected and examined for the presence of *Staphylococcus* species. As the surface sampling methods used may be applicable for other bacteria, non-staphylococci bacteria were also identified in some samples.

## Methods

### The experimental study

#### Bacterial strains for spiking of surfaces

A MSSA subsp. *aureus* Rosenbach (ATCC 29213) strain and a MRSA 50A247 strain were used in the study. Strains were grown on SaSelect agar (SA; Bio-Rad, Solna, Sweden) at 37°C for 24 h. One loopful of colony material was subsequently inoculated in Tryptone Soya Broth (TSB; Oxoid, Basingstoke, UK) with 5% sodium chloride (NaCl; Fisher Scientific, Loughborough, UK), incubated overnight at 37°C, shaken at 100 rpm. Stock cultures of the two strains were prepared by diluting overnight cultures to an OD_600_ = 0.5. Stock suspensions containing 15% glycerol were stored at −80°C.

#### Inoculation preparation and spiking of surfaces

Bacterial suspensions for spiking of surfaces were prepared by inoculation of 1 ml of stock culture in 50 ml TSB with 5% NaCl preheated to 37°C. Suspensions were incubated on a shaker at 37°C and cells diluted to an OD_600_ = 0.5, corresponding to a concentration of approximately 10^8^ CFU ml^−1^. Prior to inoculation of the stainless steel surface of a laminar flow bench, it was disinfected with 1% Virkon® (Kembo Med, Glostrup, Denmark), rinsed thoroughly with sterile milliQ-water, and treated with UV light for 1 h. A negative control of the surface was conducted using a dipslide prior to each experiment. Following, 10-fold serial dilutions with 0.1% peptone-water (Merck, Darmstadt, Germany) of the bacterial suspensions were prepared and volumes of 100 µl inoculated onto squares of 10 × 10 cm stainless steel surface of a laminar flow bench in final surface concentrations ranging from 10 to 10^4^ CFU cm^−2^. After inoculation, the surface dried for 1 h inside the closed laminar flow bench with the air flow turned off. For each experiment, the bacterial load of the suspensions was determined in duplicate by culturing 100 µl of the dilutions on nutrient agar (NA; Oxoid, Basingstoke, UK). Colony counts were calculated after incubation for 48 h at 37°C.

#### Surface sampling methods

The ability to recover the MSSA and MRSA strains spiked on the steel surfaces was evaluated using 14 combinations of sampling methods and agars. Three types of swabs and a dipslide were used. The dipslides (VWR, Søborg, Denmark) contained plate count agar (PCA, to count bacteria in general) on one side and Baird-Parker agar (BPA, selective for staphylococci) on the other side, both sides with a surface area of 10 cm^2^. The swabs used in the comparative study were (i) the eSwab transport system (eSwab; Copan, Diagnostics Inc.), consisting of a flocked nylon swab in 1 ml of modified Amies liquid transport medium, (ii) viscose-tipped transport swabs (VWR, Copan, Brescia, Italy) in a transport tube with a sponge containing 1 ml of Amies liquid transport medium, and (iii) sterile cotton-tipped wooden shaft swabs (Selefatrade AB, Spånga, Sweden) in 1 ml of sterile saline used as transport medium.

Surface sampling with dipslides was performed by carefully pressing the agar to the surface (10 cm^2^) for 10 s with no lateral movement. After sampling on one agar side, the dipslide was turned around and the adjacent surface area was sampled using the other agar side. Sampling using swabs was conducted by rotating the swab axially and laterally in a zigzag motion over the entire 100 cm^2^ surface area. The surface sampling was performed using both dry swabs and swabs pre-moistened in their respective transport medium. After sampling, swabs were transferred to the transport medium. The efficiency to recover *S. aureus* was compared by employing both direct inoculation of the swabs on NA using the roll-plate method versus elution and subsequent plating. Elution was carried out by vigorously vortexing eSwabs and cotton swabs directly in the tubes with their respective liquid transport medium for 2 min, while the viscose swabs were transferred from the transport tubes into 1 ml of sterile saline before vortexing. Samples were plated in duplicate on NA in serial dilutions. For all sampling types, incubation was performed for 48 h at 37°C.

### The field study

#### Comparative study of samplers—desks

Dipslides and pre-moistened eSwabs were used for comparative environmental sampling on the polished wood surfaces of five office desks next to the computer keyboards. The samples were taken in the spring 2017. The desks were placed in four offices, and all used by office workers who were MSSA-positive as confirmed by nasal or throat swabs. The sampling area was 100 and 10 cm^2^ for eSwabs and dipslides, respectively. Dipslides were incubated directly, while eSwabs were eluted as described above and plated in serial dilutions on both NA and SA-agar for enumeration of total aerobic, mesophilic bacteria (total bacteria) and *Staphylococcus* species, respectively. Samples were incubated for 40 h at 37°C before identification. Between 5 and 50 bacterial isolates were identified from each sample, corresponding to isolates from 10% of the entire sample volume from the eSwabs and all isolates from the dipslides.

#### Sampling of sedimenting, airborne, and surface *Staphylococcus* species

In a combined work and bedroom in Home 1, one air sample was taken using the Six-Stage Viable Andersen Cascade Impactor (ASCI) each morning during six consecutive days during in May 2017 while the occupant was getting up, and during two afternoons while the occupant was reading. The ASCI was mounted with SA-agar and sampling was done for 12 min; the detection limit was 2.9 CFU m^−3^. The ASCI is an active size-selective sampler, which samples directly onto six agar plates with a flow rate of 28.3 litres per minute (lpm). Particles of the following sizes were sampled: Stage 1: 7.0–12 µm, Stage 2: 4.7–7.0 µm, Stage 3: 3.3–4.7 µm; Stage 4: 2.2–3.3 µm; Stage 5: 1.1–2.2 µm, and Stage 6: 0.65–1.1 µm.

Sedimenting dust was also collected from the room during the 6-day period using 3 × 2 electrostatic dust collectors (209 cm^2^ sampling area per cloth; EDC, Zeeman Alphen, Netherlands). During the first 3-day period, three EDCs were placed on the top of a desk for sampling sedimenting dust. The EDCs were replaced for the second 3-day period. Following, dust from the EDC cloths was extracted in 50 ml tubes by addition of 15 ml extraction solution (pyrogen-free solution with 0.05% Tween 80 and 0.85% NaCl) by orbital shaking (500 rpm, 30 min at room temperature). Aliquots of each suspension were plated in serial dilutions on SA-agar for enumeration and identification of *Staphylococcus* species. In total, isolates from 800 µl of the sample were identified (5% of the sample). The detection limit was 0.64 CFU cm^−2^ per day. Each day, a surface sample was taken from a new area of the top of a wooden bookcase, reported to be without frequent hand contact (height 70 cm) using a pre-moistened eSwab. Samples were plated on SA-agar as described above within 2 h of sampling. Bacteria were identified as described below. *Staphylococcus aureus* isolates were inoculated on Brilliance MRSA 2 agar plates (Oxoid) to see whether they were antibiotic resistant. For clinical isolates, MRSA-selective agar has shown a specificity of 94% (Verkade *et al.*, 2011).

#### Sampling on surfaces with frequent and infrequent/no hand contact

Pre-moistened eSwabs were used for sampling on surfaces which according to the occupants had frequent hand contact, and from surfaces which usually were without hand contact (except during cleaning). Three homes were selected for the study as they had MRSA- or MSSA-positive occupants as confirmed by nasal or throat swabs. In addition, surfaces samples were taken from surfaces with frequent and infrequent hand contact in a social room at a hospital. Each sampling area was 100 cm^2^, and adjacent squares on the same surfaces were sampled. In Home 1, samples were taken from both the periphery (with frequent hand contact, *n* = 3) and the centre of a large wooden dining table (with infrequent hand contact, *n* = 3) (height 75 cm; diameter 140 cm) present in a living room. Samples were also taken from the innermost part of the top of a wooden bookcase (without hand contact, *n* = 3) (height 70 cm) present in a combined bed and workroom. The house had four occupants, and the family bred fancy chickens and pigeons as a hobby. In Home 2, samples were taken from eight areas on a floor cabinet described to be without hand contact (height: 113 cm) and from eight squares on a coffee table (height 51 cm) with hand contact. The home had two occupants and a cat. In Home 3, three samples were taken from a laptop computer, below the keyboard where the user typically rests their hands, and another three samples were taken from a metallic lamp (without hand contact) all in a combined work- and bedroom. The home had one occupant. The home samples were taken in September and November 2019. In the social room at the hospital, samples were taken in triplicate on a PVC wire channel (height 1 m) and on a lacquered hand rest, respectively, in December 2019. The eSwabs were eluted as described previously and plated in serial dilutions on SA-agar for enumeration and identification of *Staphylococcus* species. Samples were incubated for 40–50 h at 37°C and subsequently isolates from 100 µl of the sample were identified.

#### Identification of bacteria

Bacterial isolates from the environmental samples were identified using matrix-assisted laser desorption-ionization time-of-flight (MALDI-TOF) mass spectrometry (MS). A Microflex LT mass spectrometer (Bruker Daltonics, Bremen, Germany) was used for the analysis and spectra were analysed using Bruker Biotyper 3.1 software with the BDAL standard library. Bacterial isolates were prepared using the extended direct transfer methods (Madsen *et al.*, 2018). A bacterial test standard (BTS; Bruker Daltonics) was used to calibrate the instrument. Identification was performed on all bacteria from the environmental samples.

### Data analysis

#### Calculation of LOD_50_

In accordance with NordVal (Nordval, 2018), a 50% limit of detection value (LOD_50_) was calculated on the qualitative data in order to evaluate the performance of the different sampling methods. Calculations of LOD_50_ were based on the Spearman–Kärber method and express the *S. aureus* concentration (CFU cm^−2^ surface) that corresponds to a 50% probability of a positive result when using the sampling method. Calculation of LOD_50_ requires a spiking level giving a 100% positive response. In cases where none of the spiking levels gave such a response, a spiking level 10 times the highest level giving a partial positive response was assumed to give a 100% response.

#### Calculation of sampling sensitivity and efficiency

Each method was determined based on the quantitative recovery data of *S. aureus* as previously described (Dolan *et al.*, 2011) using the following equation: SE = I/(n × A) , where SE is the sensitivity, *I* is the experimentally determined number of *S. aureus* (CFU) inoculated onto the test area, *n* is the mean number of CFU recovered from the test area, and *A* is the test area in cm^2^. For each of the spiking levels, the SE was expressed as a geometric mean (GM) concentration (CFU cm^−2^) based on a minimum of four repetitions. If SE for a sample was below the LOD a concentration corresponding to 0.25 × LOD was allocated and used for calculation of GM. The sampling efficiency was calculated from the number of *S. aureus* (CFU) recovered using each sampling method as a percentage of the experimentally determined number of *S. aureus* (CFU) inoculated onto the test area.

#### Statistical analysis and data presentation

Bacterial species in surface samples are presented as CFU cm^−2^ or as relative abundance (%). Bacterial species in air samples and sedimented dust are presented as relative abundance (%). All data are presented in heat maps or bubble charts. For the heat maps, red represents the highest concentration within a sample type and environment. For the bubble charts, the size of each point is dependent on the concentration of bacteria. For values below the LOD_50_ is used for calculating the averages or GM, however if all samples are negative no averages or GMs were calculated.

Bacterial concentrations sampled from the desk surfaces using the different sampling methods were log transformed to approximate normal distribution. One-way analysis of variance performed in R version 3.5.3 was used to determine the statistical significance between total bacterial counts on desk surfaces found using (i) the different sampling kits (eSwabs versus dipslides) and (ii) different agar types (SA, NA, BPA, and PCA). Bacterial genus, species, and *Staphylococcus* richness (number) found using the different sampling kits and agar types were compared by pairwise comparison in SAS statistical software version 9.4 (SAS Institute, Cary, NC, USA). The bubble plots were made in R using ggplot2 (Wickham, 2016). The concentration of bacteria identified as *Staphylococcus* spp. as well as the total of all identified bacteria as affected by agar type and places of sampling were studied using General Linear Models in SAS. Results with *P* values of < 0.05 were considered statistically significant. Finally, the relative standard deviation (RSD) for replicate measurement of concentrations of *Staphylococcus* spp. was calculated, to compare replicate samples from surfaces with or without hand contact.

## Results

### The experimental study

#### Comparative study of samplers—spiked surfaces

The quantitative recovery results of MRSA and MSSA from artificially inoculated surfaces using dipslides, eSwabs, viscose swabs, and cotton swabs are presented in Table 1. Based on endpoint detection, pre-moistened eSwabs and viscose swabs in combination with dilution plating, and direct inoculation of pre-moistened cotton swabs were found to be the most efficient surface sampling methods for MSSA resulting in endpoint detection of 100 CFU cm^−2^ in one out of four samples. The use of pre-moistened eSwabs in combination with dilution plating was also found to result in the best recovery of MRSA from surfaces with an endpoint detection of 10 CFU cm^−2^. Using the swabs in dry form with subsequent dilution plating, MSSA and MRSA were not recovered in any of the tested concentrations. Neither was MSSA recovered using dry swabs for direct inoculation on agar.

**Table 1.** Qualitative recovery (endpoint detection) and 50% LOD_50_ of MRSA and MSSA from artificially spiked surfaces using different sampling methods. A test surface area of 10 and 100 cm^2^ was used for dipslides and swabs, respectively. Swabs were tested in a dry and pre-moistened form using both direct inoculation on agar and serial plating after elution.

Spiking concentration (CFU cm^−2^)	No. of positive samples/total no. of samples													
	Dipslide		Eswab				Viscose swab				Cotton swab			
	BPA	PCA	Pre-moist_DI_	Pre-moist_Dil_	Dry_DI_	Dry_Dil_	Pre-moist_DI_	Pre-moist_Dil_	Dry_DI_	Dry_Dil_	Pre-moist_DI_	Pre-moist_Dil_	Dry_DI_	Dry_Dil_
MSSA ATCC29213														
10^4^	4/4	4/4	3/4	8/8	0/4	0/4	3/4	8/8	0/4	0/4	3/4	6/8	0/4	0/4
10^3^	3/4	3/4	0/4	8/8	-	-	0/4	3/8	-	-	0/4	2/8	-	-
10^2^	1/5	1/5	0/4	2/8	-	-	0/4	2/8	-	-	1/4	0/8	-	-
10^1^	0/4	0/4	0/4	0/8	-	-	0/4	0/8	-	-	0/4	-	-	-
10^0^	-	-	-	-	-	-	-	-	-	-	-	-	-	-
LOD_50 CFU_ per cm^2^	917	917	1.2 × 10^4^	621	3.2 × 10^4^	3.2 × 10^4^	1.2 × 10^4^	1.4 × 10^3^	3.2 × 10^4^	3.2 × 10^4^	8.62 × 10^3^	8.62 × 10^3^	3.2 × 10^4^	3.2 × 10^4^
LOD_50 CFU_ per test area	9.2 × 10^3^	9.2 × 10^3^	1.2 × 10^6^	6.2 × 10^4^	3.2 × 10^6^	3.2 × 10^6^	1.2 × 10^6^	1.4 × 10^5^	3.2 × 10^6^	3.2 × 10^6^	8.6 × 10^5^	8.6 × 10^5^	3.2 × 10^6^	3.2 × 10^6^
MRSA 50A247														
10^4^	4/4	4/4	4/4	8/8	1/4	0/4	2/4	4/8	2/4	0/4	4/4	2/8	1/4	0/4
10^3^	1/4	2/4	4/4	4/8	0/4	-	2/4	0/8	1/4	-	1/4	1/8	0/4	-
10^2^	0/4	0/4	0/4	1/8	-	-	0/4	1/8	0/4	-	1/4	0/8	-	-
10^1^	-	-	0/4	2/8	-	-	0/4	0/8	-	-	0/4	0/8	-	-
10^0^	-	-	-	0/8	-	-	-	-	-	-	-	-	-	-
LOD_50_ CFU per cm^2^	2.3 × 10^3^	1.7 × 10^3^	860	1.0 × 10^3^	2.3 × 10^4^	3.2 × 10^4^	8.6 × 10^3^	1.4 × 10^4^	1.2 × 10^4^	3.2 × 10^4^	1.7 × 10^3^	1.9 × 10^4^	2.3 × 10^4^	3.2 × 10^4^
LOD_50_ CFU per test area	2.3 × 10^4^	1.7 × 10^4^	8.6 × 10^4^	1.0 × 10^5^	2.3 × 10^6^	3.2 × 10^6^	8.6 × 10^5^	1.4 × 10^6^	1.2 × 10^6^	3.2 × 10^6^	1.7 × 10^5^	1.9 × 10^6^	2.3 × 10^6^	3.2 × 10^6^

-, not analysed; BPA, Baird-Parker agar; DI, direct inoculation of swab on agar using roll-plate method; Dil, elution from swab and dilution plating; PCA: plate count agar.

When expressing the method sensitivities as LOD_50_, pre-moistened eSwabs in combination with dilution plating and dipslides with PCA/BPA were found to be the best for MSSA. For MRSA direct inoculation of pre-moistened eSwabs was found to give the best recovery followed by the use of pre-moistened eSwabs with dilution plating, dipslides with PCA, and pre-moistened cotton swabs with direct inoculation (Table 1).

The sensitivity and sampling efficiencies of the methods calculated based on the quantitative results are shown in Table 2. For both MSSA and MRSA, the most sensitive method was generally found to be the eSwabs in combination with dilution plating requiring a bacterial surface concentration ranging from 5.3 to 8.6 CFU cm^−2^ for MSSA and from 0.3 to 100 CFU cm^−2^ for MRSA to produce a positive result. The only exception to this was at a spiking level of 100 CFU cm^−2^ where a similar sensitivity was found using the viscose swabs with dilution plating.

**Table 2.** Sensitivity (CFU cm^−2^) of the various sampling methods for recovery of MSSA and MRSA from artificially inoculated surfaces. The sensitivity is expressed as GM values based on a minimum of four repetitions.

Spiking concentr ation (CFU cm^−2^)	Sensitivity of sampling method (CFU cm^−2^)																											
	Dipslide				eSwab								Viscose swab								Cotton swab							
	BPA		PCA		Pre-moist_DI_		Pre-moist_Dil_		Dry_DI_		Dry_Dil_		Pre-moist_DI_		Pre-moist_Dil_		Dry_DI_		Dry_Dil_		Pre-moist_DI_		Pre-moist_Dil_		Dry_DI_		Dry_Dil_	
	GM^*a*^	GSD^*b*^	GM	GSD	GM	GSD	GM	GSD	GM	GSD	GM	GSD	GM	GSD	GM	GSD	GM	GSD	GM	GSD	GM	GSD	GM	GSD	GM	GSD	GM	GSD
MSSA ATCC 29213																												
10^4^	96	2.8	130	4.3	323	18	5.3	3.1	BD	-	BD	-	5275	5.1	114	3.4	BD	-	BD	-	1822	27	66	22	BD	-	BD	-
10^3^	186	5.8	155	11	BD	-	8.2	2.4	-	-	-	-	BD	-	66	2.1	-	-	-	-	BD	-	114	1.4	-	-	-	-
10^2^	102	1.4	102	147	BD	-	8.6	2.0	-	-	-	-	BD	-	9.5	3.4	-	-	-	-	BD	-	BD	-	-	-	-	-
10^1^	BD	-	BD	-	BD	-	-	-	-	-	-	-	-	-	-	-	-	-	-	-	BD	-	-	-	-	-	-	-
10^0^	-	-	-	-	-	-	BD	-	-	-	-	-	-	-	-	-	-	-	-	-	-	-	-	-	-	-	-	-
Sampling efficiency (%)^*c*^	0.08		0.10		0.01		0.16		BD		BD		˂0.01		0.02		BD		BD		0.02		0.01		BD		BD	
MRSA 50A247																												
10^4^	147	1.5	236	5.9	98	2.0	4.7	17	4604	1.8	BD	-	1848	5.2	220	18	1338	8.0	BD	-	606	4.1	225	27	5437	1.4	BD	-
10^3^	1354	2.5	746	4.4	163	3.1	100	1.4	BD	-	-	-	805	1.8	BD	-	175	1.4	-	-	957	1.4	119	1.0	BD	-	-	-
10^2^	BD	-	BD	-	BD	-	9.0	1.2	-	-	-	-	BD	-	9.0	1.3	BD	-	-	-	77	1.8	BD	-	-	-	-	-
10^1^	BD	-	BD	-	BD	-	0.3	1.7	-	-	-	-	BD	-	BD	-	BD	-	-	-	BD	-	BD	-	-	-	-	-
10^0^	-	-	-	-	-	-	BD	-	-	-	-	-	-	-	-	-	-	-	-	-	-	-	-	-	-	-	-	-
Sampling efficiency (%)	0.07		0.05		0.01		3.41		BD		BD		˂0.01		0.09		<0.01		BD		˂0.01		0.16		˂0.01		BD	

-, not analysed; BD, below detection limit; BPA, Baird-Parker agar; DI, direct inoculation of swab on agar using roll-plate method; Dil, elution from swab and dilution plating; PCA: plate count agar.

^*a*^Geometric mean; if sensitivity (SE) for a sample was below the LOD, a concentration corresponding to 0.25 × LOD was allocated and used for calculation of GM.

^*b*^Geometric standard deviation.

^*c*^Mean percentage sampling efficiency per test area calculated based on the highest spiking concentration (10^4^ CFU cm^−2^).

### The field study

#### Comparative study of samplers—desks

**Figure 1.**
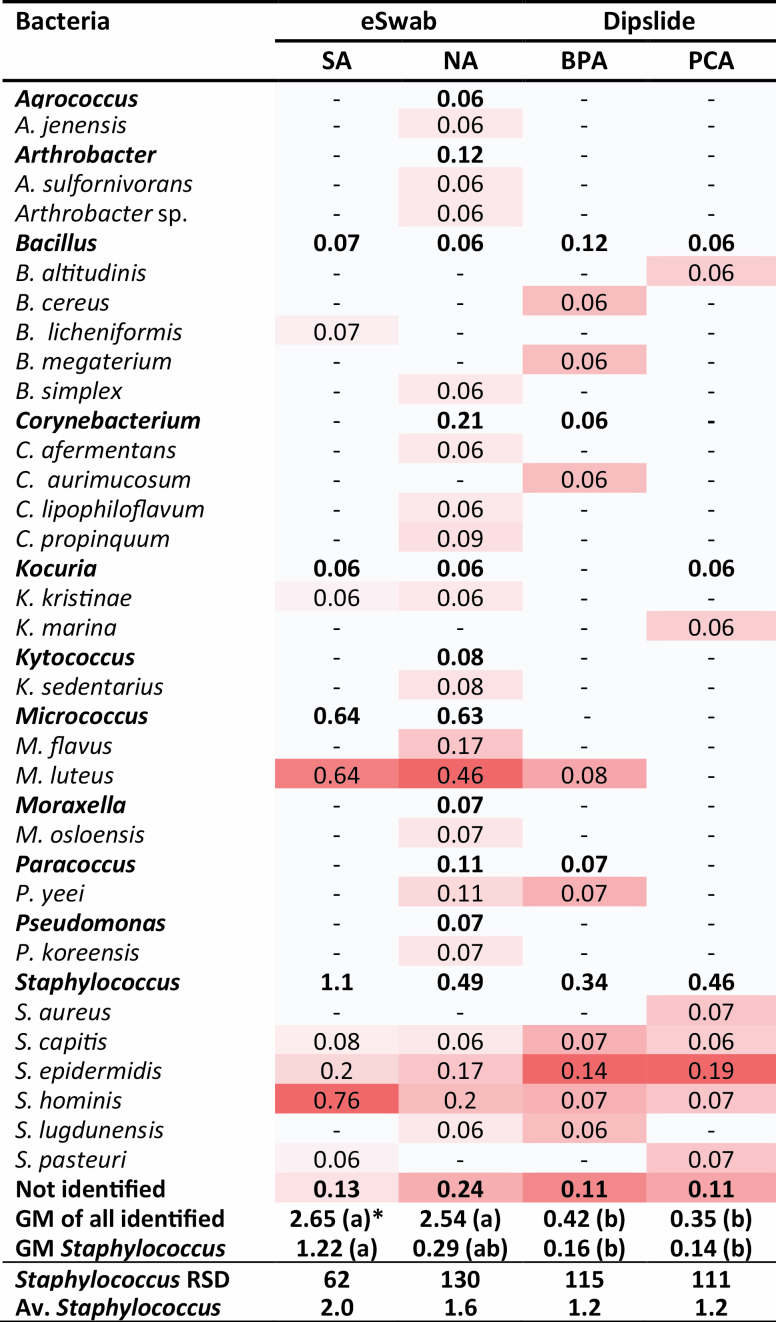
Heat map indicating the difference in concentration (GM of CFU cm^−2^, *n* = 5) of bacterial genera and species identified from five desks using eSwab with SA and NA and dipslides with BPA and PCA. _a_Concentrations in the same row with the same letter are not significantly different on a 95% significance level. -, BD; Av., average; BPA, Baird-Parker agar; NA, nutrient agar; PCA, plate count agar.

Sampling from the five desks using the pre-moistened eSwabs with dilution plating on SA and sampling and cultivation on the dipslides with BPA resulted in GM concentrations of 2.7 [range: 0.9–6.5] and 0.4 [range: 0.2–1.2] CFU cm^−2^, respectively. Sampling using pre-moistened eSwabs with dilution plating on NA and dipslides with PCA resulted in GM concentrations of 2.8 [range: 1.4–5.0] and 0.6 [range: below detection limit (BD)–2.3] CFU cm^−2^, respectively. Overall, significantly higher bacterial concentrations on desks were found with eSwabs than dipslides (*P* = 0.002). The bacterial diversity identified on the desks with the different samplers and agar types is presented in Fig. 1. As the eSwab samples on an area of 100 cm^2^ and the dipslide an area of 10 cm^2^ only 10% of the entire sample volume of the eSwabs were cultivated on NA and SA to allow direct comparison between the two sampling methods. *Staphylococcus aureus* (MSSA) was found after sampling with dipslides on PCA and only on one of the five desks tested and in a low concentration (0.3 CFU cm^−2^). No differences were found in the richness of *Staphylococcus* species between eSwabs and dipslides (*P* = 0.42) and agar types (*P* = 0.71). Both agar type (*P* = 0.0003) and desk (*P* = 0.0001) were found to have a significant effect on the measured concentration of *Staphylococcus* spp. with SA resulting in higher measured concentrations than NA, BPA, and PCA.

#### *Staphylococcus* in the air, settled dust, and on surfaces

Post sampling in the combined work and bedroom using the ASCI for airborne bacteria, *Staphylococcus* species were present in all the six studied size fractions with most S*taphylococcus* spp. present in the fraction with particles of 7.0 µm or more (see Supplementary Fig. S1, available at *Annals of Work Exposures and Health* online edition). Most species were found in several particle size fractions (data not shown). *Staphylococcus aureus* (MSSA) was found in two of eight samples (in size fractions: 1, 3, and 5 of the ASCI), and in concentrations of 2.9 and 5.9 CFU m^−3^. A considerable overlap was found in *Staphylococcus* species present in the air, sedimented dust, and surface samples, but some species constituted a lager fraction in one sample type than in another. MSSA was found in one of six surface samples (0.20 CFU cm^−2^) and in three of six samples of sedimenting dust (all three: 0.64 CFU cm^−2^). The ASCI had a higher RSD between repeated sampling than the EDC samples. Eleven species were found in both air, sedimented dust, and surface samples, and *S. capitis* was the species found in highest concentration on the surfaces (see Supplementary Fig. S2, available at *Annals of Work Exposures and Health* online edition) with an average of 8.0 CFU cm^−2^.

#### Concentrations of *Staphylococcus* species on surfaces with high and low hand contact frequency

**Figure 2.**
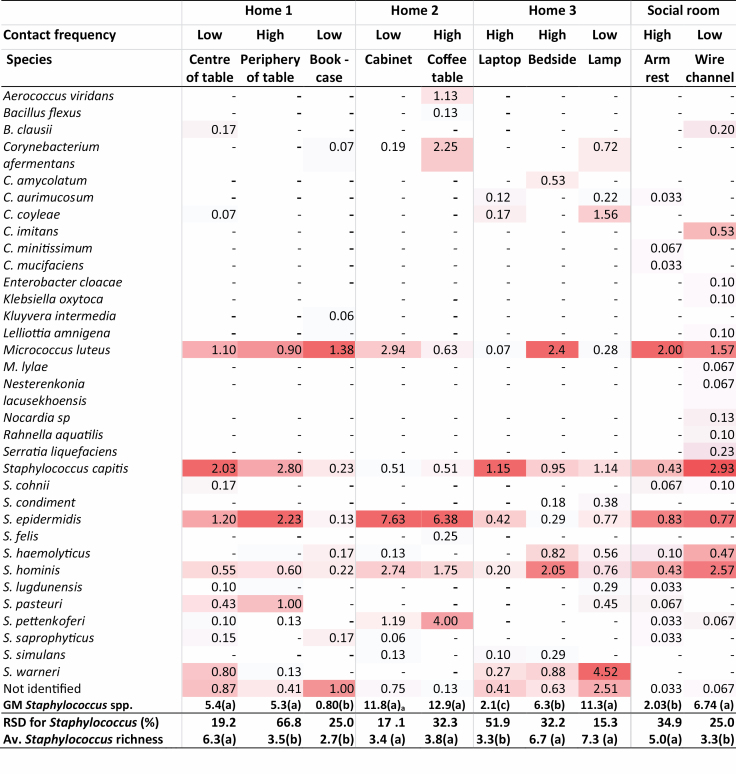
Heat map showing the concentrations (average of CFU cm^−2^) of bacterial species identified from two surfaces within three surfaces within two rooms in Home 1 (*n* = 3), one room in Home 2 (*n* = 8), three surfaces in Home 3 (*n* = 3), and two surfaces in the social room in the hospital (*n* = 3) using eSwab with SA (in the autumn and winter, 2019). _a_Concentrations and richness in the same row in the same home with the same letter are not significantly different on a 95% significance level. -, BD; Av., average; RSD, relative standard deviation for *Staphylococcus* concentration.

Using replicated sampling by swabbing with eSwabs on multiple adjacent squares on surfaces in the home environment and a social room at a hospital showed a higher RSD for concentrations of *Staphylococcus* spp. for surfaces with frequent hand contact i.e. the coffee table and the armrest than for surfaces identified to be infrequent hand contact. The *Staphylococcus* richness was highest at the centre of the dining table, the bedside, and the lamp. The *Staphylococcus* concentrations were significantly different between the locations, but the differences were not associated with the level of hand contact (Fig. 2).

In total, 13 *Staphylococcus* species were found. The species found in the highest concentrations in a home was typically found on all surfaces. Thus in Home 1, *S. capitis* and *S. epidermidis* were found in all samples. In Home 2, *S. epidermidis* (floor cabinet: 7.63 CFU cm^−2^, RSD = 12.5%; coffee table: 6.38 CFU cm^−2^, RSD = 34.7%) and *S. hominis* (floor cabinet: 2.6 CFU cm^−2^, RSD = 49.7%; coffee table: 1.75 CFU cm^−2^, RSD = 54.7%) were found in all surface samples. In Home 3, *S. warneri*, and in the social room *S. capitis* and *S. hominis* were found in all surface samples (Fig. 2). In addition to *Staphylococcus* 12 other genera constituting 20 species were found with *Micrococcus luteus* present on all surfaces, and *Corynebacterium* present with a high species richness.

## Discussion

Transmission of *S. aureus* can occur via contact with contaminated environmental surfaces (Landers *et al.*, 2010). To provide knowledge about potential transmission routes and to enable implementation of procedures to reduce transmission it is crucial to have sensitive methods which can be used to examine for surface contamination to obtain knowledge about potential transmission routes and in relation to assessing the efficacy of cleaning and disinfection procedures. In the present study, we, therefore, evaluated different sampling strategies for recovery and quantification of MSSA, MRSA, and other *Staphylococcus* species from surfaces.

Overall, the use of pre-moistened eSwabs in combination with dilution plating was found to be the most promising method for both quantitative and qualitative recovery of MRSA and MSSA with a sensitivity in the lowest detected spiking level of 0.3 and 8.6 CFU cm^−2^ for the studied MRSA and MSSA isolates, respectively. This is congruent with a previous study that determined the sensitivity of pre-moistened eSwabs to be 0.6 and 2.0 CFU cm^−2^ for recovery of MRSA from a mattress and a bench surface, respectively (Dolan *et al.*, 2011). In contrast to our study, however, they employed a pre-enrichment step of the samples prior to dilution plating. Pre-enrichment has been shown to improve the rate of detection of MRSA (Landers *et al.*, 2010; McAllister *et al.*, 2011) as it may allow stressed organisms to recover from e.g. dry surfaces and enter growth phase prior to plating on selective media (Landers *et al.*, 2010). However, such an approach is not ideal for QMRA, as it requires knowledge of the exact bacterial load present on the surface of interest. Even though the sampling methods in this study do not include a pre-enrichment step, the use of pre-moistened eSwabs still results in a method sensitivity corresponding to that found previously (Dolan *et al.*, 2011). Hence, for QMRA purposes the sampling method with pre-moistened eSwabs described here could be a good method.

On the spiked metal surface, the sensitivity of pre-moistened eSwabs was found to be approximately 10-fold higher than that of pre-moistened cotton and viscose swabs. This difference in performance between the swab-based methods can be attributed to differences in the characteristics of the swab material. The cotton and viscose swabs are generally produced by weaving the fibres around a wooden or plastic shaft while the eSwabs are produced as a one-piece construction by spraying short nylon fibres directly onto a solid, moulded plastic shaft in an electrostatic field causing the fibres to be arranged in a perpendicular brush-like structure. According to the manufacturer, this process creates a highly absorbent thin layer of fibres with an open structure. Furthermore, the eSwabs should allow for easy elution as these swabs do not contain an absorbent core to entrap the sample material. Higher liquid absorption has been documented for flocked nylon and cotton swabs compared with rayon (viscose) swabs (Moore and Griffith, 2007; Harry *et al.*, 2013). We found pre-moistened cotton swabs to result in a lower recovery of *S. aureus* than pre-moistened eSwabs and, to some extent, viscose swabs when used in combination with dilution plating. This is likely caused by poor release of bacteria from the cotton fibres which swell when wet, resulting in separation of the cellulose rings in which bacteria can be trapped (Moore and Griffith, 2007). The higher recovery of eSwabs compared with cotton and viscose swabs found in our study is in concordance with a previous study (Moore and Griffith, 2007).

The medium used for the pre-moistening and elution step may also influence the binding and release of bacteria from the swabs. In the present study, eSwabs and viscose swabs were pre-moistened in modified Amies liquid transport medium comprising an inorganic phosphate buffer, calcium and magnesium salts, and sodium chloride with a reduced environment due to the presence of sodium thioglycolate (pH 7.3–7.8). The cotton swabs were moistened using sterile saline without any buffering components (pH 6.8–7.2). This difference in composition of wetting solutions may potentially influence adhesion/detachment of the bacteria to/from the swabs. A previous study reported that the type of wetting solution affected the detachment of *S. aureus* from a steel surface with a significantly greater bacterial removal when using swabs pre-moistened with Ringer-based solutions compared with a non-nutrient, phosphate-buffered, neutralizing rinse solution (Copan SRK rinse solution) (Moore and Griffith, 2007). Furthermore, adhesion of *S. aureus* to both hydrophobic polystyrene and hydrophilic glass surfaces has been shown to be affected by the pH of the medium with significantly lower attachment to the surfaces at pH 8.0 than at pH 6.0 (Mafu *et al.*, 2011). The slightly higher pH of the Amies medium compared with that of the saline solution may, therefore, increase detachment of the bacteria from the steel test surfaces during swabbing, as well as improve bacterial release during the subsequent extraction step by reducing adhesion to swabs and walls of transport tubes. Thus, the Amies medium may contribute to the better recovery of MRSA and MSSA found with eSwabs than with cotton swabs. Although Amies liquid medium was also used for pre-moistening of viscose swabs in the present study, the sensitivity of viscose swabs was still found to be lower than that of eSwabs similarly pre-moistened in Amies medium. In addition to the obvious differences in swabbing materials, this discrepancy may be due to using a sterile saline solution as extraction liquid for the viscose swabs rather than the Amies transport medium. This was done as the viscose swab transport system used in this study is designed for direct incubation using the roll-plate method with a transport tube that contains a sponge that soaks up the liquid medium. However, this feature also makes the system incompatible with dilution plating where it is necessary to extract the bacteria into a liquid medium. The additional step of also transferring viscose swabs into a new tube with a sterile saline solution may lead to loss of bacteria prior to extraction and dilution plating.

The use of dry swabs was compared with the use of pre-moistened swabs, as dry swabs could potentially be preferable since some surfaces may be incompatible with the liquid buffer used to pre-moisten the swabs, such as electronic equipment or fragile textiles. However, our results demonstrate that using dry swabs results in a poor recovery of both MSSA and MRSA, at least for smooth steel surfaces, regardless of the type of swab used. These findings are in congruence with a previous study (Landers *et al.*, 2010). Consequently, the use of dry swabs should if possible be avoided for environmental surface sampling. In contrast, for nasal sampling wet swabs seem not to be superior to dry swabs (Hagiya *et al.*, 2013). In addition to the recovery efficiency, the prices of the surface samples could also be a parameter to consider in the selection of sampler. For the samplers in this study we paid 0.94 EUR per dipslide, 1.39 EUR per Eswab, 0.61 EUR per sterile viscose swab, and 0.25 EUR per cotton swab in 2017, and the cotton swabs did not include transport tubes and liquids.

For all the sampling methods in the present study, the recovery efficiencies from the artificially inoculated surface were found to be low with recovery efficiencies below 3.4%. Poor efficiency of surface swabbing methods is indeed an acknowledged problem. Our findings are in congruence with those of Moore and Griffith who reported the recovery efficiency of *S. aureus* from a dry surface using pre-moistened cotton, flocked nylon, and rayon (viscose) swabs in combination with dilution plating to be 1.4, 0.47, and <0.47%, respectively (Moore and Griffith, 2007). Moreover, Obee *et al.* (2007) found the efficiency of pre-moistened cotton swabs to recover MRSA from dry surfaces to be below 3.9% after direct swab inoculation using the roll-plate method. A higher recovery rate of MRSA from smooth surfaces has previously been reported for contact plates compared with swabbing (Obee *et al.*, 2007). Desiccation caused by drying the inoculum on the surfaces prior to swabbing may, however, cause stress or damage to the cells resulting in a loss of viability (Domon *et al.*, 2016; Esbelin *et al.*, 2018). This could explain the poor recovery found both with swabs and dipslides in our study. This possibility is supported by the fact that previous studies have documented a significantly higher recovery of *S. aureus* from wet surfaces compared with dry surfaces (Moore and Griffith, 2007; Obee *et al.*, 2007). Alternatively, *S. aureus* may have formed a biofilm like structure as seen on dry hospital surfaces (Ledwoch *et al.*, 2018) which may not be efficiently sampled with the tested methods.

Although surface sampling using dipslides and other types of contact plates is an easy and fast method of sampling as it does not require any subsequent sample preparation, their use is limited to flat surfaces and can only be used for sampling of a small surface area (10 cm^2^). Consequently, the contamination level observed with the dipslides may be more biased with higher sampling variance compared with swabs that are generally used for sampling areas that are 10 times larger. Whereas most other studies (Moore and Griffith, 2007; Obee *et al.*, 2007; Lutz *et al.*, 2013; Hogan *et al.*, 2015) have been limited to comparative sampling of surfaces artificially contaminated with a homogeneously distributed monoculture of *S. aureus*, the present study also compared the use of dipslides and pre-moistened eSwabs on ‘naturally contaminated’ desks. Our study demonstrated that both dipslides and pre-moistened eSwabs can be used in the indoor environment for sampling of smooth surfaces on which a low level of environmental bacteria is present, although in settings with high-level surface contamination the use of dipslides may be hampered by bacterial overgrowth. Furthermore, significantly higher total bacteria concentration levels and richness were found with eSwabs compared with dipslides. From this study, it is not possible to conclude whether this is related to the different agar types or the sampling methods. However, PCA and NA are quite similar with a neutral pH and a content of peptone, and BPA and SA are both for selective sampling of *Staphylococcus*. Despite the higher richness using eSwabs compared with dipslides, the only time *S. aureus* was detected from the office desks was in a single sample from a dipslide. As only one single *S. aureus* isolate was recovered with the dipslide this finding was likely a consequence of random variance between desk sampling sites. Overall, sampling with eSwabs in combination with dilution plating may be advantageous if a broad application is wanted as it is possible to simultaneously examine a sample on numerous types of agars, or perform other analyses as e.g. molecular analysis of the non-culturable fraction. The present study shows that other human skin-related bacteria as e.g. nine species of *Corynebacterium* as e.g. *C. afermentas* and *C*. *coyleae* were also sampled with the method.

Using replicated sampling by swabbing multiple adjacent squares on each surface in the home environment and the social room at the hospital showed a higher RSD for concentrations of *Staphylococcus* spp. for surfaces with hand contact than on surfaces without or with limited hand contact. Consequently, a greater number of samples should be taken on high-contact surfaces, to obtain a reliable measure of concentration of *Staphylococccus* spp. compared with from surfaces with infrequent contact. The *Staphylococcus* richness was not significantly different on sites with versus without frequent hand contact. *Staphylococcus* spp. were found on all studied airborne particle fractions between 0.65 and 12 µm, and these airborne bacteria may be the sources of the *Staphylococcus* spp. found on surfaces without or with low hand contact. As with any airborne particle, the sedimentation rate of airborne *Staphylococcus* depends on the particle size. In the present study about 40% of the airborne *Staphylococcus* species were present as part of particles with an aerodynamic diameter of 7–12 µm. In still air particles of these sizes with settle around 1.0–1.5 m within approx. 10 min (Popendorf, 2019).

Although the concentrations and richness of *Staphylococcus* species on the desks in the offices were lower than on surfaces in the home environments and the social room, the different environments were dominated by the same species. Thus, the following three species were found in most of the surface and air samples and in the greatest concentrations: *S. epidermidis*, *S. hominis*, and *S. capitis.* The species *S. epidermidis* is often found in the armpits and nostrils, *S. hominis* in the armpits, and *S. capitis* in the face and scalp, while *S. aureus* is mainly present in the nostrils (Kloos and Musselwhite, 1975; Becker *et al.*, 2014). In addition *S. aureus* can be found in high concentrations in exhaled breath condensate (Madsen *et al.*, 2018). A considerable overlap was found between the *Staphylococcus* species present in surface samples taken within the same environment, and between species present in air and sedimented dust samples from the same environment. For instance, *S. cohnii* and *S. warneri* were present in all sample types from Home 1, but in none of the samples from Home 2 and the offices.

In the present study a total of 66 indoor surface samples were analysed for *S. aureus*, and of these 3.0% were positive for MSSA. In a study of MRSA in public transportation, MRSA was present on at least one surface in 36% of the studied busses (Conceição *et al.*, 2013), and in eight hospitals, of which some had confirmed MRSA-positive patients, 3.3% of surface samples were positive for MRSA (Creamer *et al.*, 2014), and from 61 samples from 3 hospitals 58% were positive for MRSA (Ledwoch *et al.*, 2018). For MSSA, it has been found in 8% (in a concentration of <2.5 CFU cm^−2^) of samples taken from surfaces with hand contact from different public areas in London (Otter and French, 2009), and *S. aureus* has been found in 1 out of 25 surface samples in Polish book storerooms (Karbowska-Berent *et al.*, 2011). Thus, the different studies show a variation in share of positive MSSA and MRSA samples.

## Conclusions

In conclusion, use of dry swabs is less sensitive than use of pre-moistened swabs or dipslides. The pre-moistened eSwabs in combination with dilution plating were the most promising method for surface sampling of MSSA and MRSA on artificially inoculated smooth surfaces. This sampling approach was also found to have the broadest applicability and to be compatible with quantitative detection. Finally, the method was demonstrated to be useful for sampling of naturally contaminated smooth furniture surfaces, and using it revealed higher bacterial concentrations than using the dipslide. Application of the pre-moistened eSwabs on indoor surfaces showed the presence of 16 different *Staphylococcus* species. Sampling with the pre-moist sampler combined with plating on NA shows the presence of other genera than *Staphylococcus* as e.g. six species of *Corynebacterium*.

The RSD of surface concentrations of *Staphylococcus* spp. was higher for replicate samples on surfaces with frequent hand contact than on surfaces without frequent hand contact. Consequently, to obtain a reliable measure of concentration and the presence of *Staphylococcus* species a higher number of samples should be taken from surfaces with hand contact than from surfaces dominated by sedimented bacteria. An overlap was found between the *Staphylococcus* species present in surface samples taken within the same environment, and between species present in surface samples, sedimented dust, and air samples within the same environment. Thus air and surfaces may have the same source of bacteria and an exchange between airborne and surface bacteria seems to occur.

## Supplementary Material

wxaa080_suppl_Supplementary_Fig_S1

wxaa080_suppl_Supplementary_Fig_S2
