# Supplementary figures and images for: Evaluation of Methods for Sampling of Staphylococcus aureus and Other Staphylococcus Species from Indoor Surfaces
Source: Ann Work Expo Health. 2020 Sep 24;64(9):1020–34. doi: 10.1093/annweh/wxaa080 (PMC7750978; doi:10.1093/annweh/wxaa080)

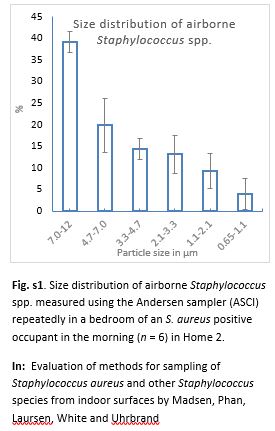

Supplement: wxaa080_suppl_Supplementary_Fig_S1 [file wxaa080_suppl_supplementary_fig_s1.jpeg]

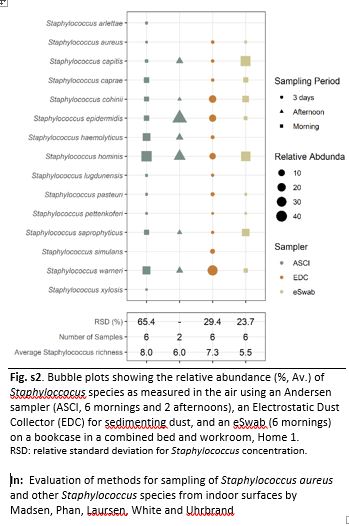

Supplement: wxaa080_suppl_Supplementary_Fig_S2 [file wxaa080_suppl_supplementary_fig_s2.jpeg]
